# Exploring pathways for building trust in vaccination and strengthening health system resilience

**DOI:** 10.1186/s12913-016-1867-7

**Published:** 2016-11-15

**Authors:** Sachiko Ozawa, Ligia Paina, Mary Qiu

**Affiliations:** 1 Division of Practice Advancement and Clinical Education, UNC Eshelman School of Pharmacy, University of North Carolina – Chapel Hill, Chapel Hill, NC 27599 USA; 2 Department of International Health, Johns Hopkins Bloomberg School of Public Health, Baltimore, MD 21205 USA

## Abstract

**Background:**

Trust is critical to generate and maintain demand for vaccines in low and middle income countries. However, there is little documentation on how health system insufficiencies affect trust in vaccination and the process of re-building trust once it has been compromised. We reflect on how disruptions to immunizations systems can affect trust in vaccination and can compromise vaccine utilization. We then explore key pathways for overcoming system vulnerabilities in order to restore trust, to strengthen the resilience of health systems and communities, and to promote vaccine utilization.

**Methods:**

Utilizing secondary data and a review of the literature, we developed a causal loop diagram (CLD) to map the determinants of building trust in immunizations. Using the CLD, we devised three scenarios to illustrate common vulnerabilities that compromise trust and pathways to strengthen trust and utilization of vaccines, specifically looking at weak health systems, harmful communication channels, and role of social capital. Spill-over effects, interactions and other dynamics in the CLD were then examined to assess leverage points to counter these vulnerabilities.

**Results:**

Trust in vaccination arises from the interactions among experiences with the health system, the various forms of communication and social capital – both external and internal to communities. When experiencing system-wide shocks such as the case in Ebola-affected countries, distrust is reinforced by feedback between the health and immunization systems where distrust often lingers even after systems are restored and spills over beyond vaccination in the broader health system. Vaccine myths or anti-vaccine movements reinforce distrust. Social capital – the collective value of social networks of community members – plays a central role in increasing levels of trust.

**Conclusions:**

Trust is important, yet underexplored, in the context of vaccine utilization. Using a CLD to illustrate various scenarios helped to explore how common health and vaccine vulnerabilities can reinforce and spill over distrust through vicious, reinforcing feedback. Restoring trust requires a careful balance between eliminating vulnerabilities and strengthening social capital and interactions among communication channels.

## Background

Demand and utilization of healthcare systems is driven by the strength of the health system itself. In the traditional context, strong healthcare systems are defined by factors such as human resource capacity, financing, information systems, and governance, and together, their ability to provide adequate service coverage and quality of care [1]. Perhaps equally important as a determinant in the demand and utilization of services, however, is trust in the healthcare system by the population it is intended to serve. Trust is commonly understood as an expectation held by an individual that the word, promise, written communication or actions of another can be relied upon [2]. Trust is an important component of social capital – the collective value of social networks of community members – and may unlock community capabilities in support of greater health [3]. Trust plays a vital role in health systems interventions where the entire arrangement is largely relational and involves an element of risk, and where the motives, intentions, and future actions of healthcare providers, community members, or the health system on which the individual depends, may not be certain [4].

Trust is a critical determinant of demand for services, and plays an essential role in user-provider interactions, which are at the center of the healthcare system. On one hand, trust in healthcare providers has been associated with increased healthcare access, treatment adherence, continuity of care, quality of care and even self-reported health status [5, 6]. On the other, weakening health systems give rise to mistrust [7]. From a systems perspective, the community level trust or the aggregation of individuals’ trust is important [8]. It contributes to the development of resilient healthcare systems, which are able to withstand major shocks and disruptions, to quickly adapt to changing circumstances, and to maintain high utilization and demand over time.

In particular, trust at both the individual and the community level plays a significant role in the demand for and utilization of vaccines, a mainstay of public health. Routine immunizations are given by healthcare providers to healthy children to prevent diseases that are becoming exceedingly rare, playing an important part in maintaining and improving population level health and eradicating diseases [9]. Building and sustaining trust in vaccination is important for uptake as successes of prevention are less visible than for treatment. As countries aim to reach high immunization coverage levels, trust has become increasingly critical across entire populations to ensure effective rates of coverage for herd immunity to protect vulnerable and immunocompromised individuals in the community [10]. Health systems must not only ensure that vaccines are safely and adequately delivered, but that information about vaccines and their delivery are also correctly relayed through the variety of information sources that communities are exposed to.

To date, insufficient research has been conducted on the dynamics that underlie how trust is developed, maintained, and lost over time, in relation to health systems and the demand for health services including vaccines [11]. These dynamics are complicated by the fact that trust is not constant over time and space, can be reinforced or disrupted at any given moment, and may take a long time to rebuild. Understanding these dynamics can provide insights into how disruptions to health systems can affect trust, and how rebuilding trust can contribute to the future resilience of health systems and communities.

Systems approaches are increasingly being used in health systems research to promote more holistic understandings of the inter-linkages and pathways of connected systems. Specifically, systems dynamic modeling has been recently applied to better define and understand these dynamics in Uganda [12, 13]. While the qualitative and quantitative system dynamics models published thus far on immunization acknowledge trust as a dynamic variable, they do not distinguish between the trust in the health system and trust in vaccination, therefore assuming that they act in similar ways. However, this may not be the case on the ground, where immunization program managers could struggle with consequences of trust arising from both vaccination and health system sources, which could exacerbate the challenges associated with these systems, particularly in low-resource settings. Furthermore, they generally do not specify how trust is conceptualized (i.e. at the individual or at an aggregate level) and assume that the sole source for trust is the effectiveness of a health center and that there is linear relationship between health center effectiveness, trust, and demand for immunization [13]. Not teasing out the various factors that can create feedback and unintended consequences along this path prevents the exploration of other potential factors through which trust can be built and re-built. To date, none of the existing models for immunization or other services [14] have focused on understanding the pathways used to rebuild trust.

In this paper, we propose to tease out the relationships between the health system, the immunization system, and trust, in order to better illustrate the determinants for building and maintaining trust in vaccination from a systems perspective. We explore the pathways for rebuilding trust in health systems based on three scenarios, which are rooted in some of the key disruptor or vulnerabilities to immunization systems (e.g. large scale epidemics or an anti-vaccine communication campaign, respectively). The scenarios we present provide an opportunity to begin the discussion around leverage points, such as community capabilities and resilience to counter the identified vulnerabilities.

## Methods

Systems dynamics provides an opportunity to examine systemic delays, feedback loops, different policy scenarios, and related intended and unintended consequences in response to exogenous shocks (such as changes in demand for services or epidemics) [13, 15]. System dynamics contains both quantitative and qualitative approaches (i.e. stock and flow diagrams, and causal loop diagrams, respectively), which can be implemented in more or less participatory ways. Building upon earlier work by Rwashana et al. [12–14], we use causal loop diagramming as a platform for a more in-depth exploration of trust. By constructing a causal map of both the determinants of trust and how trust affects eventual uptake of services, it becomes possible to see the various pathways in which trust is both developed and lost.

### Literature review

In order to capture the latest evidence on trust, immunization systems, and related challenges, we first conducted a literature review on trust in health systems, trust in vaccination, and on systems dynamics models incorporating trust in healthcare settings, in low and middle-income countries. Specifically, we conducted a rapid critical literature review of measures of trust in the health system in three major databases (PubMed, Health and Psychosocial Instruments and PsycINFO) and sought additional articles in Embase using key terms such as ‘trust’ and ‘health system;’ ‘trust’ and ‘vaccine’ or ‘immunization;’ ‘trust’ and ‘systems dynamics;’ and ‘vaccine’ and ‘hesitancy’. We also used articles on trust and health that were identified by the lead author in previous reviews. In addition to the peer-reviewed literature, we explored the grey literature, particularly to help us identify indicators that might be regularly collected and that we could use to define the variables included in the model. Wherever possible, we tried to build upon literature from low and middle income countries, rather than high income settings. Findings from the literature review were used to develop relevant variables and feedback loops in the model, as well as directionality of changes between variables. These findings also helped us define and refine the scenarios developed to illustrate our model.

### Model identification & iterative model development

We identified the following domains: trust in vaccination, trust in health systems, health and immunization system readiness, positive and negative communication arising from community sources and more broadly from sources outside the community (i.e. media, government), and utilization of both vaccines and the health system. Our main outcome of interest was the vaccine-avertable disease burden.

We began the development of the diagram based on the fundamental assumption that trust in vaccination is influenced by system readiness (or effective service delivery), but not directly. Instead, we propose that trust arises from the experience obtained by utilizing services, as well as from the messages that are transmitted [7]. We propose that the source of the message matters especially for vaccine uptake, where trusting relationships may affect behaviors [16]. Therefore, we make the distinction between messages about vaccines from within the community level (i.e. peers, community leaders, health care workers), as well as from outside (i.e. media, government). This captures trust in individuals delivering health care separate from institutional trust in the health system. We also propose that trust in the health system (i.e. medical organizations) can predict vaccination behavior [17]. While immunization systems may be a component of the health system, we have isolated the functions of immunization programs from the rest of the health system in this analysis in order to examine the interlinkages. In specifying the connecting pathways between immunization and health system levels, we assumed that trust in vaccination is causally related to trust in the overall health system and that they influence each other through a reinforcing feedback loop – i.e. if trust in the overall health system increases, trust in vaccination would also increase and vice versa.

In developing the initial causal loop diagram (CLD), we used the recommended notation from the systems dynamics literature to mark polarity, directionality of causal relationships, and feedback [15, 18]. For illustration purposes, we colored “red” the arrows with negative polarity and “blue” the articles with positive polarity. We used the dotted, “green” arrows to depict relationships of which we considered uncertain and whose polarity was highly sensitive to exogenous influences. We explored the various forms that the uncertain arrows could take through the scenario analyses described below. Uncertain loops are labelled with “U”, reinforcing loops with “R”, and balancing loops with “B.” Loops are numbered for reference purposes.

After the development of the initial model, the authors met regularly to discuss and refine the model over time. We ensured consistency in the model by exploring the various pathways in depth through uses trees, causes trees, and review of loops from key variables. Through this process we were able to check and re-check our assumptions and to ensure that the causal pathways were consistently represented throughout the various sub-systems of the diagram.

### Scenario identification

Qualitative system dynamics models, like CLDs, typically present a snapshot of the system, while quantitative system dynamics models allow for easier representation of how a system changes over time. For this paper, we present the CLD initially developed to illustrate the various pathways through which trust can not only be gained, but also lost. To help us display how these pathways change over time, we developed and illustrated scenarios. We used existing literature to identify possible scenarios related to when the relationships identified as “uncertain” take on a particular polarity. We explored how exogenous events can lead to influences on trust, potential unintended consequences and subsequent policy implications. We developed three separate scenarios to explore this question [15, 19]. In selecting and analyzing these scenarios, we paid particular attention to the notion of resilience, specifically what factors and pathways are important in a system where trust is less vulnerable to exogenous shocks, and pathways that should be targeted by policies and programs to support the rebuilding of trust. These scenarios were selected because they illustrate common challenges faced by immunization systems in low and middle income countries and can help to (1) examine the impact of health system weaknesses on trust and utilization, (2) to understand the impact of communication channels, and (3) assess the role of social capital in trust in vaccination and health systems.

### Model validation & finalization

The models were primarily developed by the authors. We validated our model for accuracy through the input of 12 key experts. The experts were purposely selected based on their experiences with causal loop diagrams and scenario-building (3), unlocking community capabilities (2), trust (2), resilience (2), and immunization (3). The experts were requested to review our paper in order to identify missing variables or relationships, and to flag spurious or undocumented relationships. The author team completed a final revision of the model and the three scenarios based on this input. The final diagram does not label all of the feedback loops that can be traced in the diagram, but focuses on the ones that we highlight in the text.

## Results

**Fig. 1:**
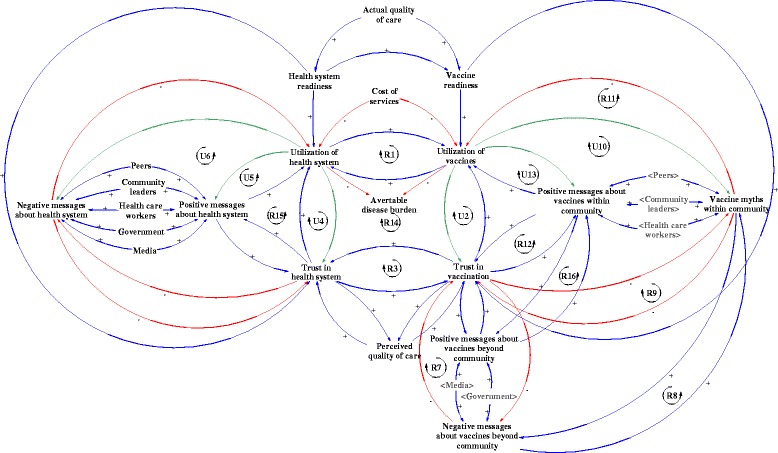
Role of trust and communication on utilizations of vaccines and the health system

Figure 1 presents the full CLD depicting the role of trust and communication in the utilization of health services and vaccines. The top part of the diagram illustrates the determinants of utilization including vaccine readiness (e.g. availability of vaccines, functional cold chain, vaccinators) and health systems readiness (overall capacity of health systems to provide general health services). In the center of the diagram is utilization of vaccines and the health system, which directly reduces the avertable disease burden. We propose that the health system and immunization system utilization are directly influenced by trust and the respective pathways of influence are illustrated in the central core of the diagram. Trust in the health system is shaped by positive and negative messages about the health system. Similarly, trust in vaccination is influenced by the messages about vaccines that can arise from both within and outside the community.

The multitude of loops illustrate the tight direct and indirect connections between the health system and the immunization system, as well as the pathways through which trust is built. We propose that health system utilization can reinforce vaccine utilization directly (see Fig. 1, R1). Similarly, the greater the trust in the health system, the greater the trust in vaccination (Fig. 1, R3). The relationship between health system utilization and trust is uncertain (Fig. 1, U4). While we posit that increased trust would certainly lead to greater utilization of services or vaccines, how the utilization experience affects trust is context-dependent. If the health system provides users with a positive experience and outcomes, then the trust in the health system would increase. However, if the experience from utilization is not positive, then increasing health system or vaccine utilization could lead to decreased levels of trust in the health system and vaccination, respectively (Fig. 1, U4 and U2). In order to build trust, the CLD shows that it is essential for utilization of health systems and vaccines to lead to positive experiences that generate positive messages following the experience of the user.

We identified further uncertainty in the relationship between health system utilization and both positive and negative messages. Ideally, increased community health system utilization would lead to increasing positive messages and decreasing negative messages. However, when the quality of health services is compromised, those utilizing services would actually contribute to propagating negative messages, rather than positive ones (Fig. 1, U5). Similarly, increased utilization of the health system causes increased trust in the health system, only in situations when the experience and outcomes are favorable (Fig. 1, U6). Following the same logic, a similar uncertainty is seen in the immunization system (Fig. 1, U10 and U13). We explore these uncertainties further through the scenarios below.

### Scenario 1: poor health systems readiness as a result of a shock to the health system

**Fig. 2:**
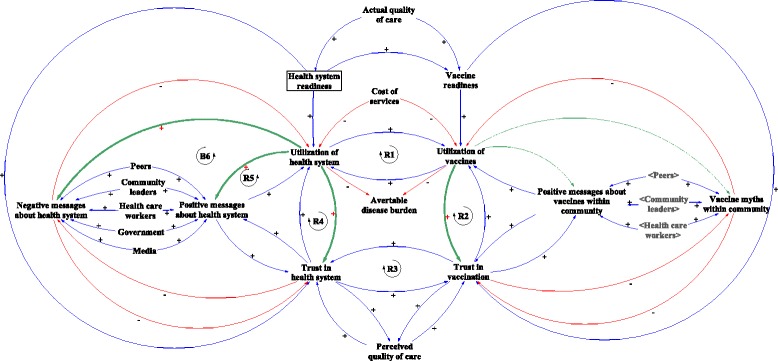
Scenario 1: Effect of poor health systems readiness on trust, communication and utilization

Weak health systems represent a root challenge to most, if not all health interventions in LMICs, including routine immunization. Critical events, such as the Ebola crisis or political conflict whose shocks ripple through the health system and ultimately affect service delivery, can exacerbate weaknesses of a health system. In Fig. 2, we illustrate how health system shocks not only influence trust in the health system, but that these issues eventually spillover into the immunization system as well, likely with a delay. In response to decreasing system readiness, a reinforcing vicious cycle develops between health care utilization and trust (Fig. 2, R4). As distrust in the health system builds, this in turn generates negative messages about the health system which again impacts utilization [20]. The CLD illustrates that all of these interactions reinforce the linkage between poor health systems readiness and low health systems utilization. Loops R5 and B6 further reinforce decreases in trust in the health system, which reinforces the decrease in trust in vaccination (Fig. 2, R3).

It is important to note the trickle down effects that the health system has on vaccination. Poor health system readiness can affect vaccine readiness, influencing utilization of vaccines. In the central part of Fig. 2, we observe that under-utilization of the health system reinforces under-utilization of vaccines and vice versa (Fig. 2, R1). Low utilization of vaccines increases distrust in vaccination (Fig. 2, R2), and distrust in vaccination reinforces distrust in the health system (Fig. 2, R3). Greater distrust in the health system reduces utilization of the health system (Fig. 2, R4), completing the reinforcing loop that links utilization with distrust, and health systems with vaccines.

While it is commonly understood that poor vaccine readiness would trigger under-utilization and distrust in vaccination, we show that distrust in vaccination can also be triggered by poor health system readiness. Even if the vaccine were available, the perceived weakness of a health system, especially following a shock, can have an effect on distrust and utilization. Even after a health system recovers from disruption, distrust tends to remain, resulting in a delay in recovery for utilization. For example, this link was observed in Sierra Leone when the Ebola virus stressed the fragile health system and degraded essential healthcare provision, resulting in a more than 20 % decrease in childhood immunization in health facilities [21]. The decrease was attributed both by lack of services from closed facilities or shortage of supplies, but also to lack of trust in the health system – i.e. community fear of going to health facilities.

Restoring health system readiness is one of the remedies to rebuilding trust. However, that alone might not be sufficient. Community-level communication and messaging – perhaps tapped through community engagement is another key pathway. Demonstrating health system responsiveness through organizing immunization campaigns, which ensure that immunizations reach children outside of the facility provides another option. This can create a temporary parallel pathway, but can help to maintain trust in vaccination, and therefore positively reinforce trust in the health system while general re-building efforts are underway.

### Scenario 2: anti-vaccine messages

**Fig. 3:**
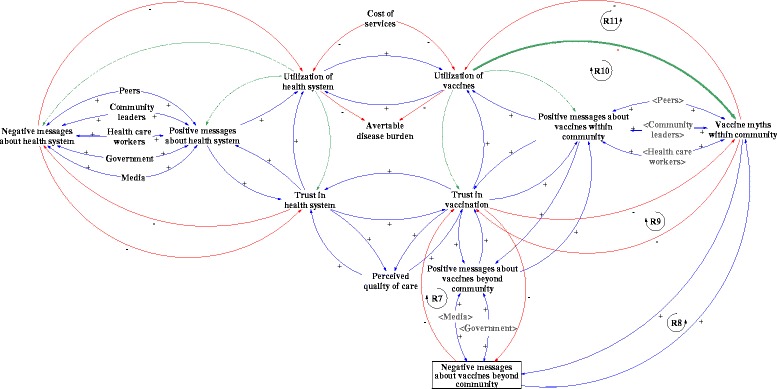
Scenario 2: Effect of anti-vaccine messages on trust and utilization of vaccines and health system

Anti-vaccine messages, myths, and negative media fuels distrust in vaccination. Negative messages about vaccines may come from beyond the community, through the media, interest groups and others in forms of rumors and stories [16]. Such messages may include suspected adverse events following immunization, conspiracies of the government and the pharmaceutical industry, fear instilled by the debunked study linking vaccines with autism, or discussions of parental rights alongside religious or naturalist beliefs. These messages are especially persistent in contexts where disease prevalence has gone down and where parents question vaccinating healthy children against something that they no longer perceive to be a relevant concern [16]. These negative messages build distrust in vaccination (Fig. 3, R7), develop vaccine myths within the community (Fig. 3, R8) and reinforce vaccination distrust (Fig. 3, R9). For example, the 2012 WHO-UNICEF Joint Reporting Form found that 19 % of un- and under-vaccinated individuals in Uganda cited lack of confidence as a factor that influenced their vaccination decision [22]. This demonstrates that lack of confidence or trust can be a significant problem even in low-income settings. Vaccine myths reduce utilization of vaccines, which further increases distrust (Fig. 3, R10). Low utilization of vaccines may continue to reinforce myths within communities (Fig. 3, R11).

There are also spillover effects from vaccines onto the health system. Distrust in vaccination can feed into distrust in the health system, leading to under-utilization of the health system. Distrust in vaccination may be triggered by poor vaccine readiness in addition to anti-vaccine messages. Therefore, improving vaccine readiness may not be sufficient to gain people’s trust and increase utilization of vaccines.

The CLD suggests two possible critical junctures to reverse or mitigate the effects of anti-vaccine messages. First, the negative messages could, in certain instances, be countered by increasing positive messages about vaccines. A recent review of the published literature suggests that dialogue-based interventions can be effective in addressing vaccine hesitancy [23]. Moreover, individually and culturally tailoring messages about vaccines is important, in order to respond to the varied types of views parents can hold [24, 25]. However, positive messages on their own will likely not counteract negative messages. Often anti-vaccine messages are more enticing than positive ones as diseases that are prevented do not make news. In addition, ‘negativity bias’ may result in anti-vaccine information having much more influence than pro-vaccine information, making it easier to lose people’s trust than it is to gain it [26]. Furthermore, recent studies suggest that positive vaccine messages or information about the risks of the disease may not always have the desired effect, reinforcing anti-vaccine sentiments among those who are already hesitant to vaccinate [27, 28]. These studies have mostly been conducted in high-income country contexts, leaving room for further exploration in low- and middle-income country settings [29, 30]. While spreading positive messages is unlikely to be a sufficient solution, it may be essential to integrate it as part of a broader package of multi-component interventions to counter reduced utilization triggered by anti-vaccine messages.

The second and equally important option is to increase the numbers of those who have positive experiences with vaccines, so that vaccine myths decreases and distrust reduces. By building positive experiences with utilization, positive messages and trust are more likely to develop than myths and distrust.

### Scenario 3: strong social capital

**Fig. 4:**
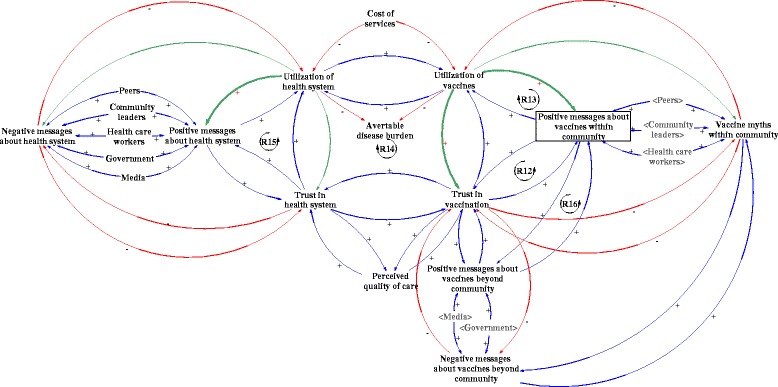
Scenario 3: Effect of social capital on trust and utilization of vaccines and health system

Where there is strong social capital, or high collective value of social networks of community members, and utilization of vaccines and the health system generates positive experiences, positive messages about vaccines within a community can be cultivated. Strength of social capital is assessed by the collective value of social networks (i.e. who people know) and the type of interactions that arise (e.g. norms of reciprocity) [31]. When communities collectively spread positive messages about vaccines, this builds trust in vaccination (Fig. 4, R12) and increases utilization of vaccines, which can yet again foster positive messages (Fig. 4, R13). This may explain how higher social capital is associated with higher immunization coverage [32]. We also recognize that the opposite might be true in certain cases. High social capital, in communities that have a poor experience with the health system or immunization system, as well as resistance to public health evidence, could lead to negative messages that are cultivated and sustained [33].

Strong positive social capital is essential for public health measures such as vaccination whereby members of the community would provide benefits to others. Vaccination protects the vulnerable in the community who cannot be immunized through indirect protection known as herd immunity, further reducing avertable disease burden [10]. Social capital in the context of positive messages about vaccines within the community can influence trust in and utilization of vaccines, which can impact trust in the health system leading to increased health system utilization (Fig. 4, R14) as well as spread positive messages about the health system (Fig. 4, R15) and affect the health of the population [34].

Social capital is important for community resilience – the greater the social capital in a context in which services are satisfactory, the less a community would be impacted by negative messages. With social capital, communities would also have a common voice to raise concerns to ensure accountability of healthcare services. The positive feedback loops (Fig. 4, R12-R15) build on community solidarity and are essential in building system resilience.

Strong social capital in a community can also spillover to neighboring communities. Positive messages about vaccines may travel beyond the immediate community to others, increasing messages beyond the community and building trust in new communities Fig. 4, R16). Such messages can unlock community capabilities to increase healthcare utilization and reduce preventable diseases. For example, strong positive social capital observed in Niger suggested that a community-based approach could improve the level of comprehensiveness and sensitivity of disease surveillance [35]. In Ethiopia, “low social capital and low economic status were associated with higher under-five mortality compared with those better-off” and those with high social capital were found to be more likely to be vaccinated [36].

## Discussion

Understanding ways to improve trust in health services including immunizations as a path to increase utilization is an underexplored area. Our analysis examined the determinants of building and re-building trust, in the context of vaccinations and the broader health system, where we hypothesized trust in vaccination and the health systems is key to maintaining resilience and avoiding vulnerabilities.

Through the development of three contrasting scenarios, we identified multiple routes through which trust in vaccination can be gained or lost. Through this analysis, we have highlighted key vulnerabilities within health systems and communication channels that are further exacerbated by major shocks to the health system or by miscommunication. This is particularly relevant as emerging infectious diseases, increasing mobility of the global population, and ongoing conflict may continue to result in large-scale health crises in the coming years that will challenge health systems, and in turn impact vaccination trust and utilization. As well, improvements to modern technology and communication channels means that the spread of information is more rapid than ever, and accessible to those who previously had no access, making it increasingly difficult to moderate and contain the spread of false information. This is critical to the future of trust in vaccination and the health system, as people increasingly turn to the internet and social media for advice and answers.

To effectively address these growing challenges, communities and their respective health systems must develop strategies that allow for the mitigation of health crises. Based on our modeling, we have identified that key areas to build resilience include:

1. Understanding major information and communication channels and how to leverage them
2. Developing effective communication strategies at all levels, to ensure that accurate information about vaccines and the health system flow from the community to government/national level
3. Supporting the development of strong health systems that can rapidly respond to crises
4. Developing high levels of positive social capital within communities on the premise that “social capital is considered not only as just protective against the impact of poverty, but also an independent predictor of child health” [36].

While the development of social capital is seemingly a vague goal, understanding the relationship between social capital, trust, and health systems is an area of importance that has not been leveraged to its full potential. The three step model described by Ogden et al. [37] to build social capital builds greater cohesion and enhances the capacity for collective action within a community, linking individuals, groups, and finally those in power together. Building high levels of social capital have been effective in post-conflict communities such as Rwanda, creating a “local environment that is generally conducive to change” [37]. Applying this model to trust in vaccinations, the creation of local groups that bring collectively minded individuals together with positive experiences can allow for greater advocacy and the voicing of information and experiences. In turn, bridging such groups across communities and with higher level policy makers can facilitate greater immunization and health system readiness, driving improved experiences, and in turn positive information.

If increased attention is not paid to trust, there is the risk that communities could lose trust and then health systems would be vulnerable to undergo shocks. In this case, the recovery will be much more difficult. As an example, the Ebola crisis displayed that the trust in the health system was so broken, due to many years of conflict from which political distrust spilled over into and amplified distrust in the health system, that there was resistance to initial attempts to curb the crisis [38]. This resistance was not overcome until greater work was initiated at the community level.

There are a number of limitations to our analysis. First, trust is not a variable that is measured easily or routinely. We therefore had to make a number of assumptions about some of the related causal relationships in the model and could not break it down further into its related components and domains in this project. While we based our analysis on existing literature, it is possible that we have omitted important variables in our analysis. Second, while we took steps to validate our model, we were only able to do so with a limited number of global experts, and there are possible linkages and perspectives that may have been missed by not being able to capture context-specific and community perspectives. Third, the current model represents a high-level representation of trust in the immunization system, which we could not validate through a context-specific example. If it were to be adapted to a particular context, the relative importance of the proposed variables may vary, and new variables may need to be introduced to accurately reflect the specific context. Fourth, due to the global representation of the model, we could not add additional distinctions that may be equally important, such as urban versus rural areas, gender roles, education, background, or socio-economic status of households/communities. Lastly, given the theoretical nature of this exercise, we had to limit the boundaries of our system to maintain feasibility of the analysis and, as such, we did not model all possible variables.

Future research should break down the concepts we present further. For example, researchers should recognize that trust is comprised of different components, manifestations [39] and domains (i.e. trust in government, trust in local leaders, trust in health providers (e.g. pediatricians [40]), medical organizations [17]) and that these can be measured and further disaggregated in a model. The relationship between the health system and public trust in government could be examined in multiple dimensions [41, 42]. The parents’ decision-making pathways pertaining to whether or not their child should be immunized and how parents’ process the information that reaches them should also be further explored, especially in low-resource settings [43]. The effect of higher incidence of vaccine avertable disease on trust and behavior change could also be examined. Future research projects should also explore social capital and their role in spreading negative messages, as well as the role that equity can play in this interaction. For example, more equitable societies tend to have higher social capital and better health [44]. Finally, in this paper, we developed dynamic causal loop diagrams to illustrate how key relationships can change under various circumstances. However, we do this using simplified pathways. It would be beneficial to apply the model in an actual implementation context with context-specific dynamics.

Based on our findings, there are a number of implications for policy makers that may help sustain successes in vaccination programs. For example, development of crisis mitigation strategies could be important to prepare communities for future health system shocks. Creation of improved communication channels relating to health systems and vaccines, appropriate to local context, would be important to build trust in vaccination and health system related information. Finally, building linkages among government, media, health care workers, community leaders and community members to build trust in information about the health system and vaccination would build more resilience.

## Conclusions

Trust is important, yet underexplored, in the context of vaccine and health systems utilization. Using a CLD to illustrate various scenarios helped to explore how health and vaccine vulnerabilities can reinforce, spill over and generate distrust through vicious, reinforcing feedback. The exploration could be deepened by adapting the CLD to a particular context, where it could also be a useful local level planning tool. Restoring trust requires a careful balance between eliminating vulnerabilities and strengthening social capital and interactions among communication channels. Systems shocks cannot all be prevented or controlled – natural calamities, disease epidemics, conflict or political turmoil happen. By deliberately focusing on and building trust and social capital, health systems could become more resilient and more apt to manage these shocks and quick to recover from them.

## Abbreviations

CLD: Causal loop diagram
WHO-UNICEF: World Health Organization – United Nations Children’s Fund
